# Supplementary material for: Association between the Right Ventricular Longitudinal Shortening Fraction and Mortality in Acute Respiratory Distress Syndrome Related to COVID-19 Infection: A Prospective Study
Source: J Clin Med. 2022 May 6;11(9):2625. doi: 10.3390/jcm11092625 (PMC9103975; doi:10.3390/jcm11092625)
Supplement: Supplementary file 1 [file jcm-11-02625-s001.zip › jcm-1706794-supplementary figure S1.pdf]

Supplementary Files

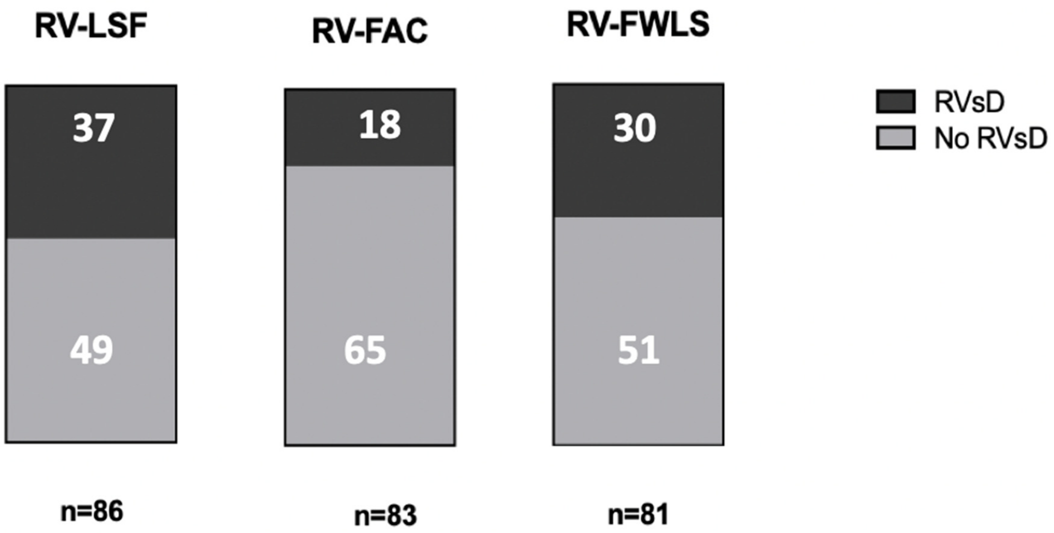

Figure S1. Legend: number of patients with RVsD according to the echocardiographic definition.

RVsD: right ventricular systolic dysfunction. **RV-LSF**: right ventricular longitudinal shortening fraction**; RV-FWLS**: right ventricular free wall longitudinal strain; **RV-GLS**: right ventricular global longitudinal strain.
